# Enhanced efficiency in plastic waste upcycling: the role of mesoporosity and acidity in zeolites†

**DOI:** 10.1039/d4sc05121a

**Published:** 2024-10-31

**Authors:** Saideep Singh, Joaquín Martínez-Ortigosa, Nuria Ortuño, Vivek Polshettiwar, Javier García-Martínez

**Affiliations:** a Department of Chemical Sciences, Tata Institute of Fundamental Research Mumbai 40005 India vivekpol@tifr.res.in j.garcia@ua.es; b Instituto de Tecnología Química, Universitat Politècnica de València – Consejo Superior de Investigaciones Científicas (UPV-CSIC) Avda. de los Naranjos s/n 46022 Valencia Spain; c Laboratorio de Nanotecnología Molecular, Departamento de Química Inorgánica, Universidad de Alicante Ctra. San Vicente-Alicante s/n 03690 Alicante Spain; d University Institute of Chemical Process Engineering, University of Alicante Carretera de San Vicente del Raspeig, s/n 03690 Alicante Spain

## Abstract

By modulating zeolite confinement and improving pore diffusion properties, addressing a significant limitation in current plastic waste upcycling methodologies is essential. In this work, we have developed mesoporous zeolites that exhibit enhanced diffusion capabilities for long-chain polymers without compromising the crystalline structure. The mesopore volume doubled from 0.14 cm^3^ g^−1^ (CBV720) to 0.28 cm^3^ g^−1^ (M720_3h_) after zeolite modification. This has enabled to overcome the inefficiencies associated with polymer diffusion in conventional zeolites, significantly advancing the catalytic conversion of plastic waste into valuable products. Catalytic pyrolysis experiments on various polyethylenes underline the superior performance of mesoporous zeolites, especially for highly branched polymer structures where degradation temperatures are reduced by 29 °C compared to conventional zeolites, highlighting the importance of pore arrangement. Detailed analysis using NH_3_-TPD and *in situ* DRIFT spectroscopy reveals the crucial role of Brønsted acid sites in enhancing degradation efficiency. The optimized mesoporous zeolite catalyst, M720_cit_, showed excellent effectiveness in reducing degradation temperatures for a wide range of daily-use plastic waste. The *T*_10_ values were significantly reduced for various plastic wastes: food packaging dropped to 208 °C (from 354 °C), plastic bottles to 349 °C (from 381 °C), and milk packets to 277 °C (from 409 °C), among others. Moreover, the well-retained microstructure of the M720 catalyst yielded a very similar product distribution despite the introduction of mesoporosity. This study not only surmounts crucial obstacles in the modulation of zeolite confinement and the enhancement of pore diffusion properties but also augments the economic and environmental sustainability of plastic waste conversion processes.

## Abstract 2

By optimizing zeolite pore confinement, polymer diffusion and up-cycling efficiency in hierarchical catalysts are significantly enhanced, reducing plastic degradation temperatures and providing a suitable route to sustainable plastic-to-fuel pathways.

## Graphical abstract

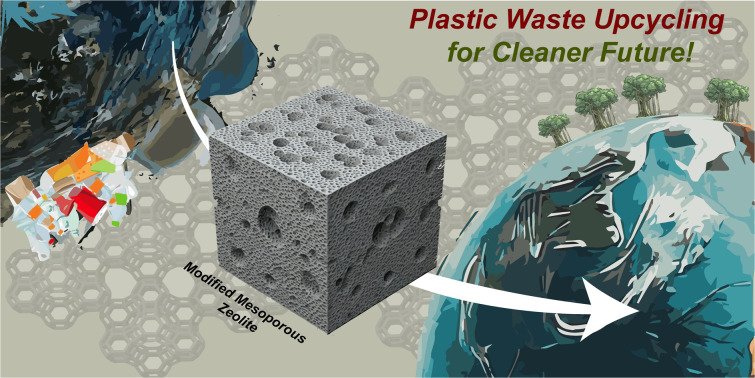

## 1 Introduction

The burgeoning environmental impact of plastic waste poses a significant challenge to ecosystems and human health. The extensive production and disposal of non-degradable plastic items have led to the build-up of plastic waste in both aquatic environments and landfills affecting wildlife and, through the food chain, human health.^1^ This crisis necessitates urgent and innovative solutions, among which plastic waste upcycling emerges as a promising strategy.^2^ Upcycling, the process of transforming waste materials into new products of higher quality or utility, offers an effective means to mitigate the environmental impact of plastic waste. New plastics can be fabricated by combining different polymer grades from upcycling using additives. Hence, unlike traditional recycling, upcycling adds value to the waste, making it a more environmentally and economically attractive option.^3^

In this context, zeolite-based catalysts have garnered significant attention due to their strong acidity, porous structures, high surface area, and thermal stability. These characteristics make zeolites popularly used in catalysing chemical reactions as an acid catalyst for cracking,^4^ isomerization,^5^ and alkylation.^6^

Despite their potential, existing zeolite-based catalysts face several challenges that limit their widespread application in plastic waste upcycling. One of the primary issues is the efficient diffusion of plastic polymer chains into the zeolite pores.^7,8^ The size and complexity of plastic polymers often hinder their ability to penetrate the micropores of zeolites, thus reducing the efficiency of the catalytic process. In this sense, introducing mesoporosity in zeolites seems promising to address problems faced in microporous zeolites.^9,10^ Among them, the surfactant templating technique has proved to be an efficient way to introduce well-defined, tunable, and highly interconnected mesopores in FAU-type zeolites, while maintaining the key properties of conventional zeolites, *i.e.* crystalline structure, hydrothermal stability, and strong acidity.^11,12^ Due to their superior and improved characteristics for the conversion of bulky molecules compared to the traditional zeolites, the surfactant-templated zeolites have been successfully transferred to industry.^13,14^ However, the confinement effects within the zeolite structure, crucial for catalytic activity, are not fully understood and optimized in the context of plastic waste upcycling with different branching and different types.

Medium pore-size zeolites, such as MFI-type, are diffusively restricted for the conversion of bulky compounds.^15,16^ Hence, the catalytic cracking of the polymer (end chain cracking) mainly occurs in the external surface of the crystals or in the pore-mouth of the channels, giving rise to a high proportion of light hydrocarbons (C_2_–C_5_).^15^ However, the presence of large pore zeolites or the introduction of mesoporosity in traditional zeolites enhances the diffusion of larger plastic fragments, allowing them to reach the micropores and undergo a secondary type of cracking, yielding the formation of a mixture of middle and light hydrocarbons.^17–21^ In this context, a close analysis of the literature shows that the two main factors that determine the performance of a catalyst are the Brønsted acid sites (BAS) and the accessibility of the molecules to the microporous structure.^22,23^ A synergy of these two parameters is the key to develop a superior catalyst. For example, Al-MCM-41, which shows excellent accessibility presents a poor performance because of weak acidity. Although many studies discuss the ability of micro/meso-porous zeolites to crack polymers, there is a debate regarding which process is dominant during catalytic cracking. Regarding the selectivity of the process, many factors influence it, such as the pore size of the zeolite, the presence of mesoporosity and the strength of those BAS. In general, the acid strength impacts the cracking capacity of the materials: stronger BAS will result in terminal cracking to form light hydrocarbons, while medium-light strength will be selective to the formation of longer chains or waxes.^17,21^ The modulation of a potential secondary cracking is affected by the nature and distribution of the BAS and the diffusion process of the molecules leaving the catalyst where the textural properties, *i.e.* improved accessibility, play a determining role.^18^

This work addresses these critical challenges by exploring innovative strategies to modulate the confinement and pore diffusion properties of zeolites at very low temperatures (<280 °C) where the thermal cracking was not feasible.^15^ Through a comprehensive study, we enhanced the efficiency of FAU-type zeolite catalysts in plastic waste upcycling, paving the way for more sustainable and economically viable waste management solutions.

## 2 Results and discussion

### Synthesis and material characterization

CBV720 zeolite (FAU-type) was used as a starting material to prepare the materials studied in this work. Mesoporous zeolites were successfully prepared by surfactant templating as described elsewhere.^24,25^ Two different treatment times 3 h (M720_3h_) and 24 h (M720_24h_) were used, giving rise to materials with superior textural properties. More specifically, the mesopore volume doubled from 0.14 cm^3^ g^−1^ (CBV720) to 0.28 cm^3^ g^−1^ (M720_3h_), as shown in Table 1.

**Table 1:** Chemical composition and main textural properties of the materials prepared

Material	Si/Al_EDX_	*V*_micro_ (cm^3^ g^−1^)	*V*_meso_ (cm^3^ g^−1^)
CBV720	16.5 ± 2.5	0.26	0.14
CBV720_HFSi_	54.3 ± 3.9	0.22	0.20
CBV720_cit_	20.7 ± 0.8	0.25	0.16
M720_3h_	14.6 ± 1.0	0.22	0.28
M720_HFSi_	38.3 ± 5.5	0.18	0.31
M720_cit_	21.0 ± 2.8	0.22	0.28
M720_24h_	16.6 ± 1.0	0.20	0.33
Al-MCM-41	7.5 ± 3.1	0.00	0.46

**Fig. 1:**
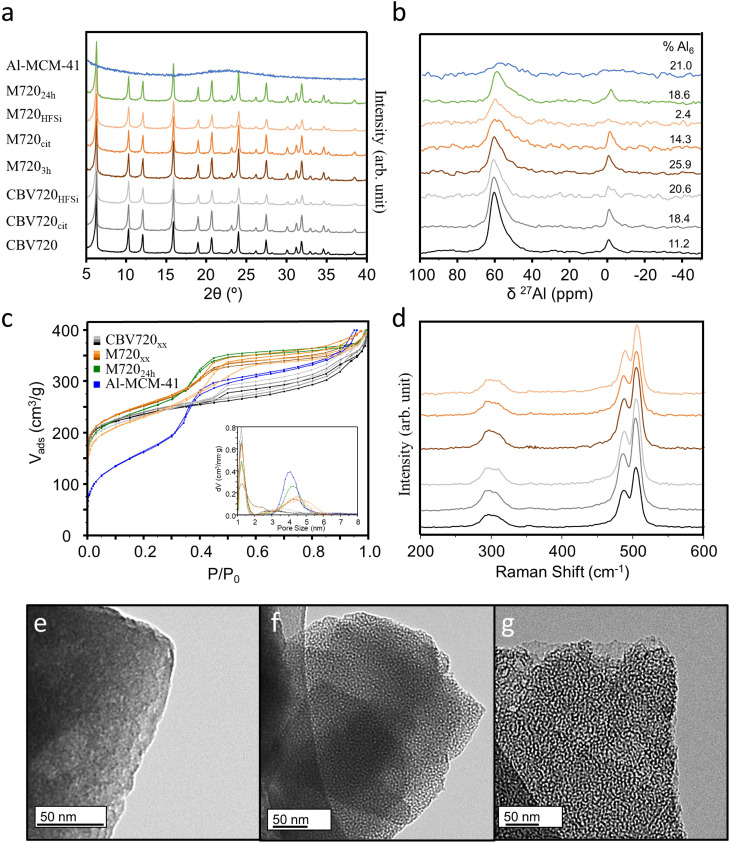
Structural characterization. (a) X-ray diffraction patterns, (b) ^27^Al MAS-NMR, (c) N_2_ isotherms, (d) Raman spectroscopy of the zeolites prepared. TEM images of (e) CBV720, (f) M720_3h_ and (g) Al-MCM-41 materials as representative of microporous and mesoporous materials.

To remove any debris material that could partially block the microporosity, samples CBV720 and M720_3h_ were treated with either citric acid (to increase the accessible Brønsted acid sites in the zeolite)^26^ or NH_4_SiF_6_ (in order to remove some extra-framework (EFAL) Al species).^27,28^ Samples were labelled as CBV720_XX_ or M720_XX_, where XX is ‘cit’ or ‘HFSi’ depending on whether citric acid or NH_4_SiF_6_ were used for treatment. The post-synthetic treatment with citric acid did not modify the micropore and mesopore volumes and the textural properties remained nearly unaltered (Table 1) as shown in Fig. 1c. However, the NH_4_SiF_6_, slightly increases the *V*_meso_ at the expense of the diminution of *V*_micro_; it is well known that the aggressive character of the reactant will be able to locally destroy part of the zeolitic framework creating bigger “holes” which are reflected in the *V*_meso_.^29^ A reference surfactant-templated material Al-MCM-41, with a similar mesopore volume but no microporosity was synthesized for comparison purposes and its properties are also given in Table 1. The mesoporosity of the surfactant-templated zeolite and Al-MCM-41 material was observed by TEM (Fig. 1e–g) confirming its narrow pore size distribution and high connectivity in contrast to the commercial material where no well-defined mesopores are able to be identified by TEM (Fig. 1e).^11^

Table 1 shows the ratio of Si/Al of all samples, obtained by SEM-EDX. After the introduction of mesoporosity neither the Si/Al ratio nor the FAU structure (Fig. 1a) was significantly affected; however, after the washing treatments with citric acid or NH_4_SiF_6_ the Si/Al ratio increased, suggesting that some aluminium was removed from the sample; as expected, this effect was more noticeable in the samples washed with NH_4_SiF_6_ causing an increase of the Si/Al from ∼14.6 to more than 38. The structural features of the washed samples were checked by pXRD and Raman spectroscopy, showing the typical pattern of FAU topology indicating that the local order typical of this structure is well preserved (Fig. 1a and d) after the post synthetic treatments.

The evolution in the nature of the aluminium atoms in the zeolites submitted to the surfactant templating and the posterior washing treatments was studied by ^27^Al MAS-NMR and the results are displayed in Fig. 1b. In all ^27^Al MAS-NMR spectra, it was possible to identify the two more common signals for zeolites, the tetrahedral Al (Al_Td_) at around 55 ppm and the octahedral (Al_Oh_) at 0 ppm. The parent material CBV720 possesses 88.8% of the aluminium atoms as Al_Td_ while the 11.2% remains in Al_Oh_. After the surfactant templating procedure, the Al_Oh_ increases to 25.9%, while the Si/Al ratio does not vary significantly, meaning that some aluminium atoms leave the framework during the creation of the mesopores and stay as the EFAL within the framework. The washing treatments affect differently when the materials are microporous (CBV720) or mesoporous (M720_*x*h_), probably due to the easier diffusion of the reactants and EFAl species along the pores and channels.^30^ The use of citric acid does not vary the Si/Al ratio but when using the CBV720 more EFAL species are created (due to dealumination) and the opposite trend is observed when washing the M720 material (due to realumination) (Fig. 1b). The use of NH_4_SiF_6_, typically used for removing EFAl species,^27^ changes significantly the Si/Al ratio and creates more EFAl species in the conventional zeolite while in the mesoporous zeolite the treatment occurs successfully and practically all the EFAL disappears. For M720, washing with NH_4_SiF_6_ predominantly removed the EFAl (Al_Oh_ = 2.4%), while washing with citric acid removed a portion of the EFAl (Al_Oh_ = 14.3%). It is important to note the broadening of the Al_Td_ signal in M720_cit/HFSi_ samples indicating that some Al is now present in the tetrahedral distorted coordination^31^ or as Al pentacoordinated.^32^

### Catalytic pyrolysis study of polyethylenes (PEs)

The pore arrangement in a zeolite plays a crucial role in determining the accessibility of its acid sites to the polyolefin reactant and intermediates, thereby impacting its overall activity.^33^ Additionally, different zeolites exhibit distinct topological and microporous structures, directly influencing their catalytic performance and deactivation (or lifetime). Consequently, any exploration of structure–activity relationships in polyolefin cracking should consider both acidity and the pore structure.^34^ In our study, different polyethylenes (PE) having different degrees of branching were used as model plastics to know the role of accessibility (well-interconnected micro/mesoporosity) for the conversion of low to highly ramified (bulky) polymers. For this, 2 mg of different zeolites were mixed with 6 mg of PEs (namely low-density polyethylene (LDPE), linear low-density polyethylene (LLDPE), and high-density polyethylene (HDPE)) in a mortar pestle. Fig. S1† shows the sample preparation steps for TG analysis. These three different types of polyethylene have various degrees of branching. The HDPE has the least branching whereas the LDPE has long branching. The thermal and catalytic degradation of the polymers were evaluated by thermogravimetric analysis (TGA). First, the adsorbed moisture from the catalyst surface was removed by heating the sample at 130 °C under nitrogen flow. After that, the mixture was heated from 130 °C to 700 °C under a nitrogen flow. The *T*_*x*_ (temperature to reach *x*% of weight loss) was determined for all samples.

**Fig. 2:**
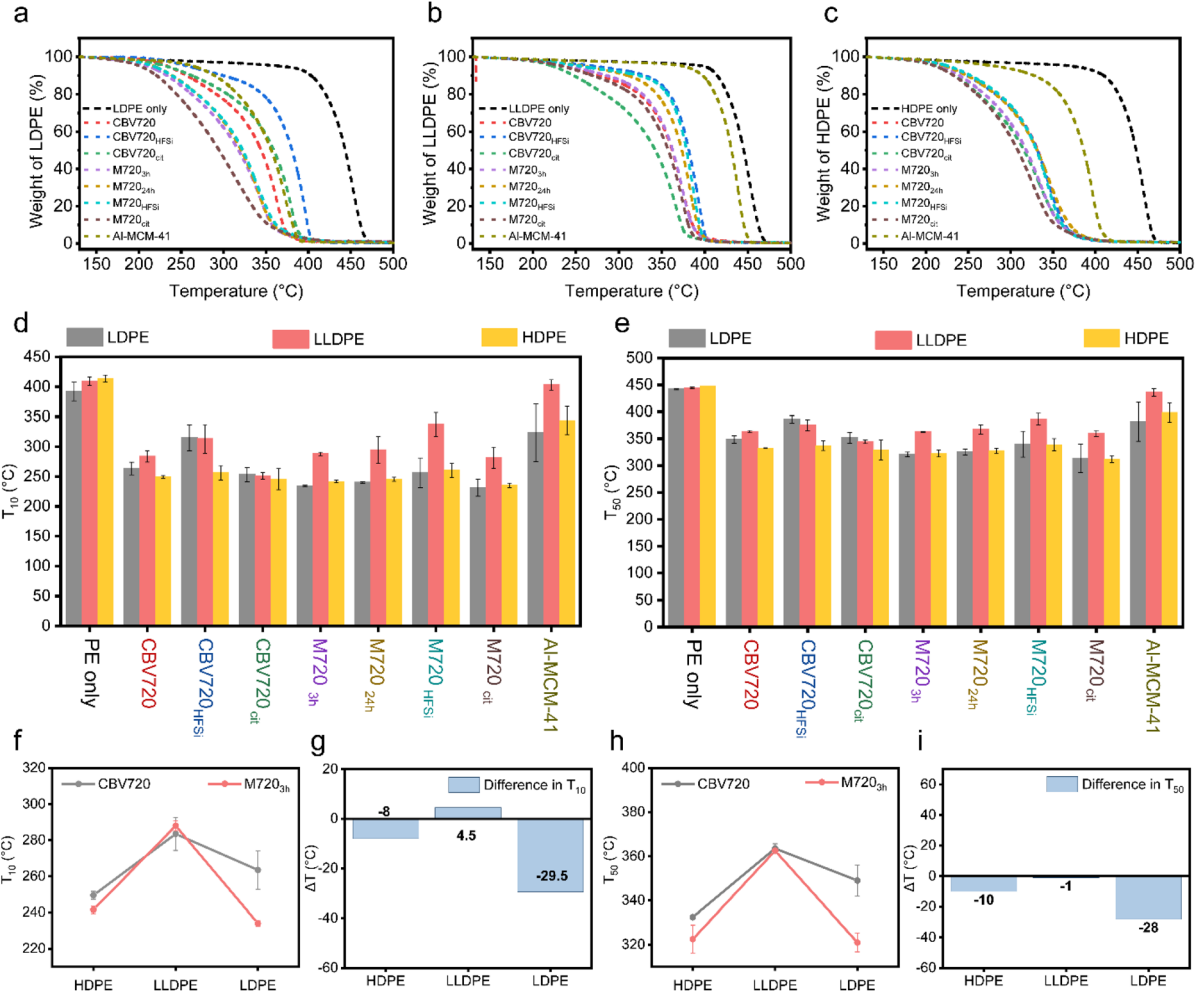
Plastic degradation study by TGA. Weight loss curve of (a) LDPE, (b) LLDPE, and (c) HDPE pyrolysis using different zeolites. (d) *T*_10_'s and (e) *T*_50_'s of the pyrolysis of different PEs. Comparison of (f and g) *T*_10_'s and (h and i) *T*_50_'s of commercial CBV720 *vs.* mesoporous M720_3h_ zeolite. Error bars: calculated from data of at least two repeated experiments. PE-polyethylene, LDPE-low-density polyethylene, LLDPE-linear low-density polyethylene, and HDPE-high-density polyethylene.

The weight loss curve for different types of polyethylenes (PEs) was plotted against the temperature (Fig. 2a–c). The addition of the catalyst to the PEs causes a significant reduction in the degradation temperature (up to *T*_10_ of 178.5 °C, see Table S1†) because of the well-known cracking activity of the strong acid sites of the zeolite.^17,35^ In all the cases, the mesoporous zeolite, *i.e.* M720_3h_, behaved better in plastic degradation than parent CBV720 (Fig. 2d and e). The contrast between the two was found to be more prominent in the case of LDPE, which has more branches. A LDPE degradation study was also conducted using different weights of the catalyst. Weight loss curves with 1.8, 2.0 and 2.2 mg of catalyst overlap with each other in both cases (Fig. S2†). Additionally, 2 mg of M720_3h_ outperformed all the different screened amounts of CBV720.

**Fig. 3:**
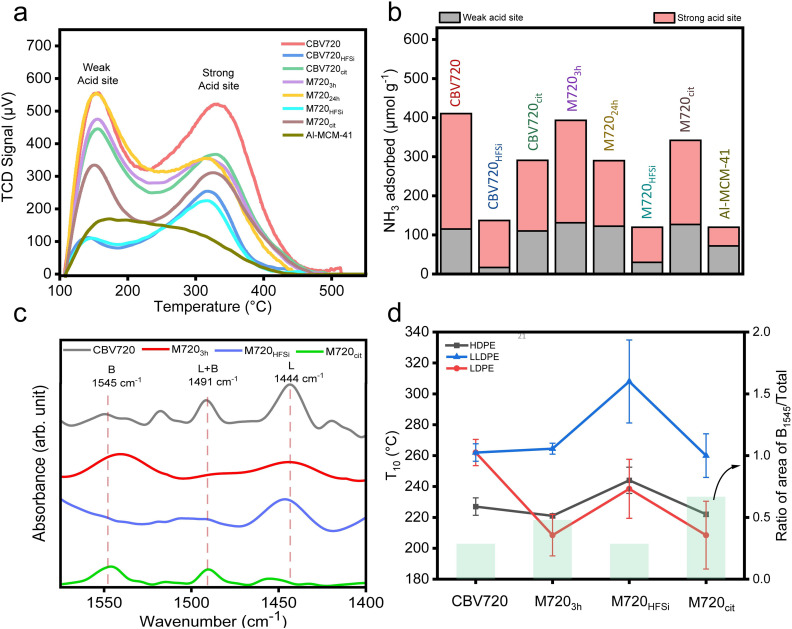
NH_3_-TPD profile and in-situ DRIFT of different zeolites. (a) The TCD signal from ammonia desorption *vs.* temperature (not normalized to grams of catalyst). (b) Comparison of total acidic sites present along with individual contribution from weak and strong acidic sites. Pyridine adsorption study. (c) DRIFT spectrum of pyridine on various catalysts after pyridine desorption at 523 K. (d) Comparison of *T*_10_'s of all the types of PE over different zeolites, and the ratio of the area of Brønsted acid peak *vs.* total.

Notably, increasing the mesopore volume, by increasing the time of surfactant templating treatment (3 h *vs.* 24 h), does not improve the capacity of the PE cracking of the synthesized micro/meso zeolites M720, pointing out that there is no relationship between the mesopore volume and the cracking capacity of the catalyst. We propose that the presence of a certain amount of mesopores is crucial to start cracking the bigger portions of plastics and then those portions can diffuse through the micropores to reach other Brønsted acid sites and continue breaking into smaller polymer fractions. This observation was evident in the comparative analysis of M720_3h_ and M720_24h_ across all three polyethylene (PE) variants where M720_3h_ behaved slightly better than M720_24h_ (Fig. 2f–i) but remained very similar. The removal of some debris alumina from the M720_3h_ sample after the citric acid treatment was found to have a positive effect on the accessibility to the BrØnsted acid sites, as revealed by Py-DRIFT and NH_3_-TPD (Fig. 3), and thus better performance during the cracking of the PE because of its superior acidic properties, as discussed in the next section.

In fact, M720_cit_ proved to be the most effective zeolite for the cracking of all types of PE. More specifically, the *T*_10_ values reduced to 231 °C ± 14 °C from 402 °C ± 1 °C when the catalyst was used along with LDPE. Mesoporous aluminosilicate (*i.e.*, Al-MCM-41) was also used in the catalytic cracking of polyolefins for comparison purposes as a benchmark material with just mesopores. The Al-MCM-41 catalysts exhibit diminished acidity, yet demonstrate improved accessibility of activity sites situated within the mesopores.^33^ Compared to M720_3h_ catalysts, the Al-MCM-41 catalysts showed higher *T*_*x*_ values. This underscores the limitation of relying solely on mesopores for comprehensive plastic cracking and highlights the key role of the presence of strong zeolite-like acidity for the proposed application.

Both the enhanced accessibility provided by the introduced mesoporosity and the strong acidity preserved in the surfactant-templated zeolites are needed for facile cracking of PE, characteristics present in M720_3h_. Deep insights into the significant advantage of precise control over pore volume and size as well as pore architecture, as determined from the N_2_-adsorption study, were obtained by plotting the difference in *T*_10_ of M720_3h_ and CBV720 (Fig. 2f–i). We found that Δ*T*_10_ remains almost the same for HDPE and LLDPE, while it decreases significantly for LDPE. It clearly showed that the zeolite M720_3h_ favors LDPE (having long branching) over HDPE (having no branching) since it has more mesopores (0.28 cm^3^ g^−1^) than CBV720 (0.14 cm^3^ g^−1^).

### Acidic characteristics of the catalyst

The acidity inherent in zeolites is a crucial factor in plastic cracking.^34^ An approach to determining the number of acidic sites involves quantifying the base molecules like NH_3_, chemisorbed onto the acidic sites present in the zeolites.^36^ The total acidity of each catalyst was determined by the NH_3_-TPD (Temperature Programmed Desorption) method, and the results are summarized in Fig. 3a and b. All the tested zeolites showed two peaks, a small desorption peak centered at approximately 150 °C, followed by a broad NH_3_ desorption peak ranging from 250 to 450 °C, indicating that the zeolitic materials featured two different types of acidic sites: weak and strong. A typical TPD spectrum of a zeolite exhibits two desorption peaks: the l-peak (at lower temperatures) and the h-peak (at higher temperatures).^37^ The l-peak, weak acidic sites, cannot be attributed to desorption from a specific type of acid site; rather, it arises from the intense ammonia saturation experienced by the acidic sites.^37,38^

Initially, ammonia molecules adsorb on Brønsted acid sites and robust Lewis sites, forming ammonium sites and ammonia-framework complexes. Incoming molecules get adsorbed on newly formed NH_4_^+^, probably by hydrogen bridging and dispersive interactions giving the l-peak. The strong acidic sites, h-peak, on the other hand, are the consequence of ammonia desorption from true acidic centres. On comparing CBV720 and M720_3h_, we found that the total acidity was comparable (410 *vs.* 393 μmol g^−1^ respectively) but there was a clear difference in the type of acidic sites present (Fig. S3†). We also found that the total acidity of the best catalyst M720_cit_ (342 μmol g^−1^), was slightly lower than that of M720_3h_ (393 μmol g^−1^). The common configuration of ammonia TPD allows us to get the total number of acidic sites in each zeolite but, it does not allow distinguishing between Brønsted acid sites (BASs) and Lewis acid sites (LASs). *In situ* DRIFT spectroscopy of various probe molecules adsorbed on zeolites has proven useful to overcome this drawback.^39^Fig. 3c presents IR spectra of the various catalysts following pyridine adsorption. In the case of studied zeolites, the peak at 1444 cm^−1^ arising from the C–C stretching vibration of the coordination-bonded pyridine complex indicated the presence of Lewis acid sites. The peak at 1545 cm^−1^, attributed to the C–C stretching vibration of the pyridinium ion (PyH^+^), showed the presence of Brønsted acid sites, and the peak at approximately 1490 cm^−1^ was assigned to pyridine adsorbed on both Lewis and Brønsted acid sites. On comparing the area under the peak assigned to the Lewis acid sites and Brønsted acid sites, we found that the ratio of the area of Brønsted acid and total acid sites was highest in M720_cit_ and lowest in M720_HFSi_. As the ratio of the area of Brønsted acid and total acid sites increases, the *T*_10_ values decrease in all types of plastics (Fig. 3d).

These results suggest that (a) M720_cit_ has primarily Brønsted acid sites together with a lower number of Lewis acid sites, and (b) higher amounts of Brønsted acid sites are responsible for better plastic degradation in our FAU-type zeolites as reported in literature, where BAS are responsible for reactions of monomolecular cracking and dehydrogenation of *n*-butane, propane and *n*-pentane *via* the carbonium-ion mechanism.^40–43^ As a conclusion, the presence of mesopores and the right proportion of BAS over LAS as in the M720_3h_ and M720_cit_ zeolites favors LDPE (having long branching) over HDPE (having no branching) or LLDPE (having medium branching) because the latter two can not easily diffuse over the micropores and be cracked.

### LDPE cracking selectivity

**Fig. 4:**
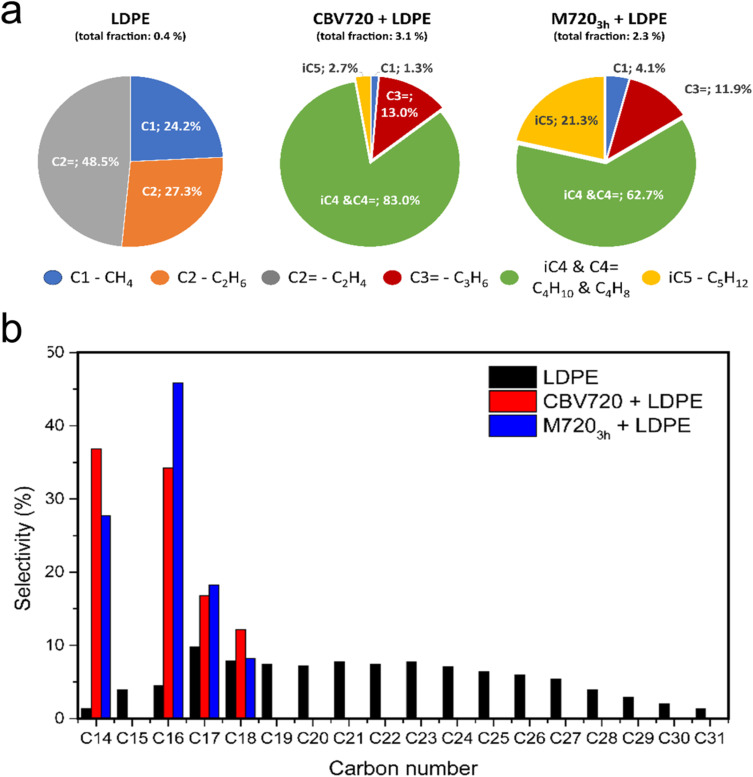
LDPE cracking profile. (a) Gas fraction and (b) non-volatile compound selectivity analysis.

The degradation process of a plastic, as discussed before, can occur *via* thermal degradation or *via* catalytic cracking, at lower temperatures. Based on the TGAs (see Fig. 2a) the experiments done in the presence of both zeolites (at around 250 °C), are completely based on catalytic cracking. The product distribution dramatically changes when catalytic cracking rules the degradation process (see Fig. 4a), while the employment of a conventional or mesoporous zeolite does not have a huge impact on the product distribution. During the thermal cracking, only C1 and C2 (both alkane and alkene) were detected, while for the experiments with zeolites CBV720 and M720_3h_ a very similar distribution was observed for both of them. We identified them as methane, propene, C4 (alkane and alkenes) and iso-pentane. Some aspects to highlight regarding the results of the analysis of the gas fraction for the catalytic cracking are: (1) the presence of methane points to a cracking mechanism which involves the presence of carbonium cations that only can take place in the BAS;^44,45^ and (2) the presence of a high proportion of mesoporosity in M720_3h_ gives rise to shorter diffusion paths enabling the production of heavier hydrocarbons, as is the case of iso-pentane, and prevents deep cracking of hydrocarbons.^46^

Fig. 4b shows the product distribution of the non-volatile fractions that was adsorbed in the resin and then extracted with organic solvents. For the non-catalyzed experiment a wide distribution is observed which covers all the C species that can be formed, from C14 to C31 pointing out that there is no control of the process.^20^ However, the presence of a catalyst, either CBV720 or M720_3h_, dramatically changes the product distribution. The products observed range from C14 to C28 for both zeolites, but the major (and quantifiable) contributions are the hydrocarbons C14, C16, C17, and C18, the smaller chains being favoured for CBV720 and the bigger ones for M720_3h_. In the non-volatile fraction analysis by GC-MS, alongside the linear hydrocarbons, a signal corresponding to the 1-alkene species was also detected, consistent with previous reports. However, the quantification of alkene compounds was not feasible due to their smaller peak area and coelution with other species, which made accurate integration challenging.^47^ In light of the results, the use of mesoporous FAU-type zeolite, which has improved textural properties and a lack of some acidity with respect to the parent commercial CBV720 zeolite, has two positive effects on plastic cracking: (1) the presence of mesopores facilitates the initial cracking of the bulky molecules of LDPE, lowering the *T*_50_ temperature by around 30 °C;^20^ and (2) despite the introduction of mesopores, associated with the presence of more Si–OH defective sites, the selectivity of the material is practically identical to that of the commercial CBV720 zeolite, highlighting the similarity between the materials after the mesopore introduction and the preservation of the FAU microstructure.

### Efficient degradation of plastic waste from daily life sources

**Fig. 5:**
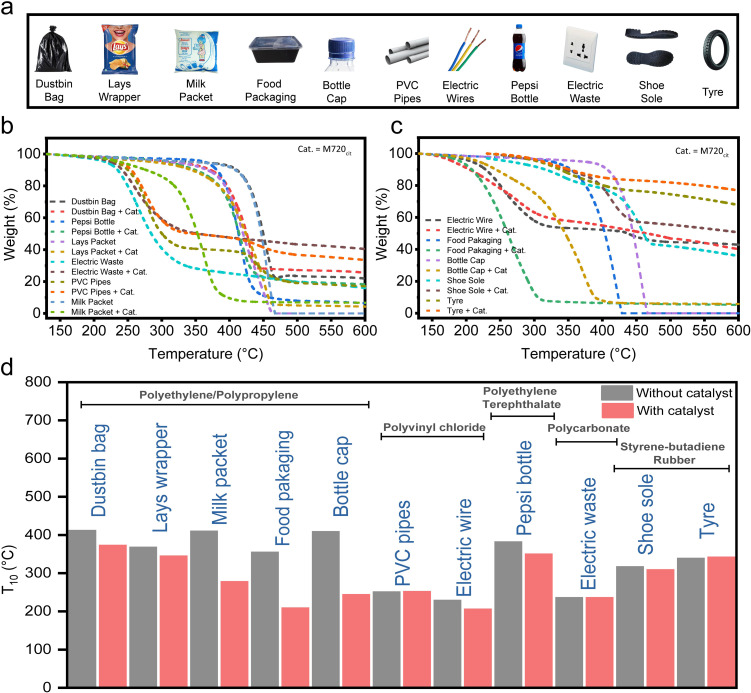
Plastic waste degradation Study. (a) Different plastic wastes collected from our surroundings. (b and c) Weight loss curves of plastic waste (6 mg each), and using M720_cit_ (2 mg). (d) Comparison of *T*_10_'s of different plastic waste used in the study.

The universality of the best-screened catalyst (M720_cit_) was studied *via* the degradation of ‘real’ plastics in daily household (see Table S2† for the composition of each daily plastic), such as food packaging, plastic bottles, milk packet, electric wire, *etc.* as shown in Fig. 5a–d. Notably, the *T*_10_ of food packaging was reduced to 208 °C, (from 354 °C), plastic bottles to 349 °C (from 381 °C), milk packets to 277 °C (from 409 °C), and electric wire to 205 °C (from 228 °C) (Fig. 5d). We found that M720_cit_ was good at degrading food packaging and bottle caps. The nominal reduction in the *T*_10_ of the plastic bottle was because, while food packaging and milk packets are predominantly made of hydrocarbons, plastic bottles are made from polyethylene terephthalate which are hard to break. (Table S2†). In summary, as shown in the model study with pure HDPE, LDPE or LLDPE, FAU-type zeolites and their mesoporous versions are a good candidate to degrade some daily-life plastics.

## 3 Conclusions

The escalating environmental impact of plastic waste on ecosystems and human health necessitates innovative solutions, with plastic waste upcycling emerging as a promising strategy. Zeolite-based catalysts, known for their unique properties, show potential in catalysing the conversion of plastic waste into valuable products. However, challenges, such as inefficient diffusion of long-chain plastics into zeolite pores, hinder their widespread application. We address these challenges by exploring innovative strategies to modulate the pore-size diffusion properties of zeolites. The study focuses on the synthesis and characterization of mesoporous zeolites using surfactant templating techniques resulting in a significant increase in mesopore volume from 0.14 cm^3^ g^−1^ (CBV720) to 0.28 cm^3^ g^−1^ (M720_3h_), improving diffusion without compromising the crystalline structure as well as in the product distribution results. ^27^Al MAS-NMR shows the evolution of EFAl, which left the framework during the creation of the mesopores after surfactant templating. The research investigates the catalytic pyrolysis of different types of polyethylenes using these zeolites. The results highlight the role of the zeolite pore architecture, demonstrating enhanced plastic degradation with mesoporous zeolites compared to conventional zeolites. The introduction of mesoporosity improves the cracking capacity of the catalyst, particularly for polyethylenes with higher branching. Insights into the acidic characteristics of the catalysts reveal the importance of acidic sites. The study employs NH_3_-TPD and *in situ* Py-DRIFT spectroscopy to quantify acidic sites and distinguish between Brønsted and Lewis acid sites. Mesoporous zeolites with the right proportion of Brønsted acid sites exhibit superior plastic degradation, emphasizing the significance of both mesoporosity and acidity. The manuscript concludes by applying the optimized zeolite catalyst (M720_cit_) to degrade various daily-life plastics, showcasing its effectiveness in reducing degradation temperatures. For example, the *T*_10_ value for food packaging decreased from 354 °C to 208 °C, for plastic bottles from 381 °C to 349 °C, and for milk packets from 409 °C to 277 °C, demonstrating its effectiveness in lowering degradation temperatures across various plastic types.

## Data availability

All data are available in the manuscript and ESI.

## Author contributions

V. P. and J. G.-M. conceived the concept, designed the experiments, and supervised the project. S. S., J. M.-O. and N. O. designed various experiments and performed the experiments. Data were analysed by S. S., J. M.-O., N. O. and V. P. The initial draft of the manuscript was penned by J. M.-O., S. S. and V. P., and was subsequently refined by the entire team.

## Conflicts of interest

There are no conflicts to declare.

## Supplementary Material

SC-015-D4SC05121A-s001
