# Supplementary material for: Enhanced efficiency in plastic waste upcycling: the role of mesoporosity and acidity in zeolites
Source: Chem Sci. 2024 Oct 31;15(48):20240–50. doi: 10.1039/d4sc05121a (PMC11575530; doi:10.1039/d4sc05121a)
Supplement: SC-015-D4SC05121A-s001 [file SC-015-D4SC05121A-s001.pdf]

Supplementary Information (SI) for Chemical Science.
This journal is © The Royal Society of Chemistry 2024

Enhanced Efficiency in Plastic Waste Upcycling: The Role of Mesoporosity and Acidity in Zeolites

Saideep Singh[1#], Joaquin Martínez-Ortigosa[2,3#], Nuria Ortuño[4], Vivek Polshettiwar*[1], Javier García-Martínez*[3]

[1] Department of Chemical Sciences, Tata Institute of Fundamental Research, Mumbai, 40005 India

[2] Instituto de Tecnología Química, Universitat Politècnica de València – Consejo Superior de Investigaciones Científicas (UPV-CSIC), Avda. de los Naranjos s/n, 46022 Valencia, Spain

[3] Laboratorio de Nanotecnología Molecular, Departamento de Química Inorgánica, Universidad de Alicante, Ctra. San Vicente-Alicante s/n, 03690, Alicante, Spain

[4] University Institute of Chemical Process Engineering, University of Alicante, Carretera de San Vicente del Raspeig, s/n, 03690 Alicante, Spain
Email: vivekpol@tifr.res.in, j.garcia@ua.es, # shared 1st authors

Table of Contents in Supplementary Information:

Figure S1. Sample preparation for TG analysis.

Figure S2. Weight loss curve of LDPE (6 mg) pyrolysis by varying the catalyst (CBV720 and $M720_{3h}$) amount to 1.8, 2.0 and 2.2 mg.

Figure S3. Ammonia TPD of (a) CBV720, (b) $CBV720_{HFSi}$, (c) $CBV720_{cit}$, (d) $M720_{3h}$, (e) $M720_{24h}$, (f) $M720_{HFSi}$, (g) $M720_{cit}$, (h) Al-MCM-41.

Table S1. T_{10} and T_{50} for degradation of HDPE, LLDPE and LDPE (6 mg) using different catalysts (2 mg).

Table S2. Different plastic wastes and their composition.

Materials and Methods

Material synthesis. Three different types of materials were used for the catalytic degradation of PE: 1) commercial CBV720 (Zeolyst) as a microporous material; 2) mesoporous M720 zeolite prepared by the surfactant templating technique[i,ii] (3h and 24h at 150 °C in steel stainless autoclaves in static conditions) which combines the existence of microporosity and mesoporosity; and 3) the well-known Al-MCM-41 material presenting just mesoporous openings. The surfactant templating technique consisted of mixing the commercial zeolite 2.5 g CBV720 with 160ml NH_3 (1.4% w/w) aqueous solution containing the CTAB (1.75 g) surfactant, followed by the hydrothermal treatment described above. Al-MCM-41 was prepared by mixing 9.3 ml TEOS (Sigma-Aldrich), 0.4 g $AlCl_3$ (Sigma-Aldrich) and 1.75 g CTAB in a 170 ml aqueous solution of NH_3 (1.2% w/w), stirring the mixture for 3 h at room temperature followed by hydrothermal treatment for 24 h at 110 °C. After the synthesis, the samples were filtered, washed with abundant distilled water and calcined at 550 °C during 6 hours.

The resulting zeolitic materials (CBV720 and mesoporous zeolite $M720_{3h}$) were treated with two different solutions 50 ml containing citric acid at 10mM (Sigma-Aldrich) or hexafluorosilicate (HFSi) at 5mM (Sigma-Aldrich) under stirring conditions at 80 °C during 2 hours. Afterwards, the suspensions were filtered and washed with distilled water and dried in an oven at 110 ºC overnight. After the post-synthetic treatments, the materials were calcined using the same program described before.

The materials will be labelled as CBV720 and M720 for the microporous and the mesoporous zeolites respectively and adding "xxx" if the zeolites were treated with citric acid (cit) or with hexafluorosilicate and Al-MCM-41.

Characterization. X-ray diffraction (XRD) patterns were collected in a powder X-ray diffractometer (Bruker AXS D8 Advance) with graphite monochromatized Cu Kα radiation at 40 kV and 40 mA. Nitrogen physisorption isotherms at −196°C were performed in an AUTOSORB-6 apparatus. The samples were previously degassed for 8 h at 250°C at 5×10−5 bar. Adsorption data were analysed by using the software QuadraWinTM (ver. 6.0) of Quantachrome Instruments. Cumulative pore volumes and pore-size distribution curves were calculated by using the DFT method (NL-DFT adsorption branch model). The total pore volume was obtained at the plateau of the cumulative adsorption pore volume plot at a relative pressure (P/P0) of 0.9. Micropore volume was determined by NL-DFT as the volume adsorbed at pore sizes < 2 nm, and the mesopore (< 10 nm) volume was calculated by subtracting the micropore volume from the total pore volume.[iii] ^{27}Al solid state MAS nuclear magnetic resonance experiments were recorded in a 500 MHz (BRUKER AVANCE DRX500) spectrometer using a π/12 of 1 µs and 1s of D1. The integration of the spectra was done by using the Bruker TopSpin 3.6.1 program. The UV-Raman spectra were obtained on a Jasco NRS-5100 dispersive Raman system with a UV-Vis laser source of 325 nm. The morphology of the samples was studied by transmission electron microscopy (TEM) images collected by using a JEM-1400 Plus microscope (JEOL, 120 kV, 0.38 nm resolution). The chemical composition was obtained by using EDX-SEM technique with a Jeol IT500HR/LA apparatus.

Pyridine DRIFT study. Around 10 mg of zeolite was taken in the alumina crucible. The sample was degassed at 450 °C for 1h under argon flow of 10 mL min-1. Sample as baseline was taken (1000 scans). Pyridine vapor was passed through the reaction chamber for 30 mins using argon flow of 10 mL min-1. Weakly adsorbed pyridine was removed under argon flow of 20 mL min-1 at 240 °C for 30 mins. DRIFT spectrum was taken (1000 scans) afterward.

Ammonia Temperature Programmed Desorption (NH3-TPD) Study. The analysis was performed using a flow-through microreactor system equipped with TCD (BELCAT Ⅱ) and coupled with Mass Analyzer. Around 45 mg of the sample was taken for analysis. First, the sample is heated to 450 °C (10 °C min-1) under argon flow of 40 mL min-1. The sample was heated at 450 °C for 60 mins to remove the adsorbed moisture on the surface of zeolites. The sample was cooled to 100 °C under argon followed by introduction of 10% NH_3/He (40 mL min^{-1}) for 30 mins. The extra NH_3 was removed by flowing pure argon gas (50 mL min^{-1}). After that, the sample was heated to 600 °C (5 °C min^{-1}) under 30 mL min^{-1} argon flow.

Feedstocks. LDPE [melt index, 25g/10 min (190 °C/2.16 kg; density, 0.925 g/mL)], LLDPE [melt index, 1.0 g/10 min (190 °C/2.16 kg; density, 0.918 g/mL)], and HDPE [melt index, 12g/10 min (190 °C/2.16 kg; density, 0.952 g/mL; Mw, 115,828 Da], were purchased from Sigma-Aldrich. Waste plastics were collected from the surroundings that are commonly used. Their compositions are given in the supplementary Table S2.

LDPE selectivity study & analysis. The pyrolysis runs were conducted using a tubular quartz reactor located inside a horizontal laboratory furnace described in detail elsewhere.[iv] The neat LDPE sample (8 mg) was tested at 415 ºC, while LDPE:Zeolite mixtures (6:2 mg) were submitted to 240 ºC and 260 ºC for the mesoporous ($M720_{3h}$) and the microporous (CBV720) zeolites, respectively. These temperatures were selected based on the results of the previously conducted thermogravimetric analysis (TGA). Typically, these kinds of studies are conducted using several grams of plastic to ensure the identification of all products formed, due to mass limitations in our experiments, our focus was on identifying the major and main products. All the reactions were stopped between 36% and 41% of conversion in order to be able to establish a fair selectivity comparison. The identified products in the gas fraction range from C1 to C5 and from C14 to C31 in the non-volatile fraction. The fraction between C6 to C13 was not identified in our experiments, pointing out that its formation is not favoured in the conditions used in this work. The gas yield was 0.4% for the experiment in the absence of catalyst, 2.3% for the $M720_{3h}$ and 3.1% for the CBV720 catalyst, results which are in line with the higher acidity of the CBV720 that results in higher proportion of light hydrocarbons.[v] For each run, nitrogen was introduced in parallel flow to the sample at a constant rate of 50 mL/min (purity of 99.9992%, S. E. Carburos Metálicos, S. A., Barcelona, Spain). The sample was placed in a quartz boat, which was introduced into the reactor at 130 °C and then heated at a rate of 3ºC/min to reach the temperatures indicated above. The compounds leaving the reactor were sampled during 20 min, beginning when the temperature was around 10 °C below the final temperature. Gases and volatile compounds: Gases and volatile compounds were collected in Tedlar® bags (Restek, Madrid, Spain) at the reactor exit. A gas chromatograph with a flame ionization detector (GC-FID, TRACE 1600 model, Thermo Fisher Scientific, Madrid, Spain) was used for the analysis of light hydrocarbons (methane to isooctane), using a TracePLOT TG-BOND Alum (30m x 0.32mm x 5μm) capillary column (Thermo Fisher Scientific, Madrid, Spain). Identification and quantification of these compounds were performed using external standard calibration with a C1-C8 hydrocarbon mix (S. E. Carburos Metálicos S. A., Barcelona, Spain). Non-volatile compounds: Amberlite® XAD-2 resin (Supelco, Pennsylvania, USA) was used to adsorb the semivolatile compounds in the flue gases. Then the resin was extracted with dichloromethane/acetone (1:1 vol.) by accelerated solvent extraction (Dionex ASE® 350, Thermo Fisher Scientific, Madrid, Spain) and the extracts concentrated to approx. 1 ml. All solvents for organic trace analysis were purchased from Merck (Germany) and are pesticide grade. Non-volatile compounds were analysed using high-performance gas chromatography (Agilent 6890 N) with mass spectrometry (Agilent 5973 N) in the SCAN mode (35–550 amu) with an Agilent HP5-MS (30 m × 0.25 mm i.d. × 0.25 μm) capillary column. Compounds were identified by comparison with the NIST mass spectral database and the identification of each alkane was confirmed by comparing the retention time with an external standard (C7-C40 Saturated Alkanes Standard (49452-U), Sigma-Aldrich, Missouri, USA).

Plastic degradation study by TGA. The analysis was performed using a Mettler Toledo TGA instrument. Plastic (6 mg) and catalyst (2 mg) were mixed in a mortar pestle, and loaded in an alumina crucible (70 μL). The mixture was first heated to 130 °C (10 °C min^{-1}) under nitrogen flow of 50 mL min-1. After staying for 30 mins at 130 °C, the mixture was heated to 500 °C (3 °C min^{-1}) and then, to 700 °C (10 °C min^{-1}) under the same nitrogen flow. A similar procedure was used for the waste plastic degradation study.

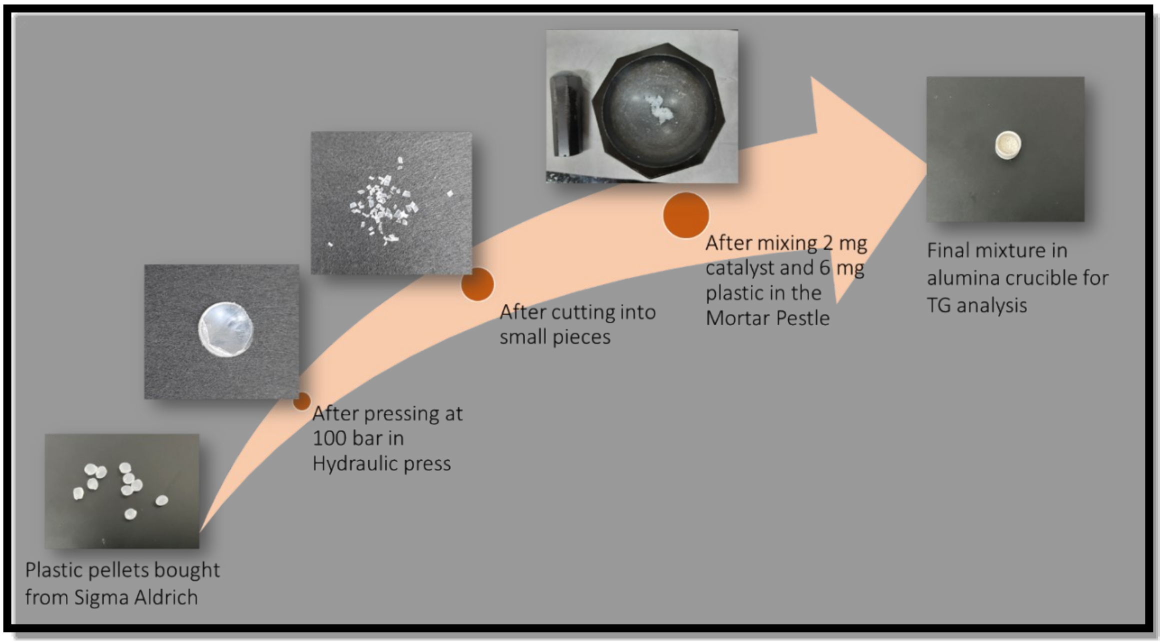

Figure S1. Sample preparation for TG analysis.

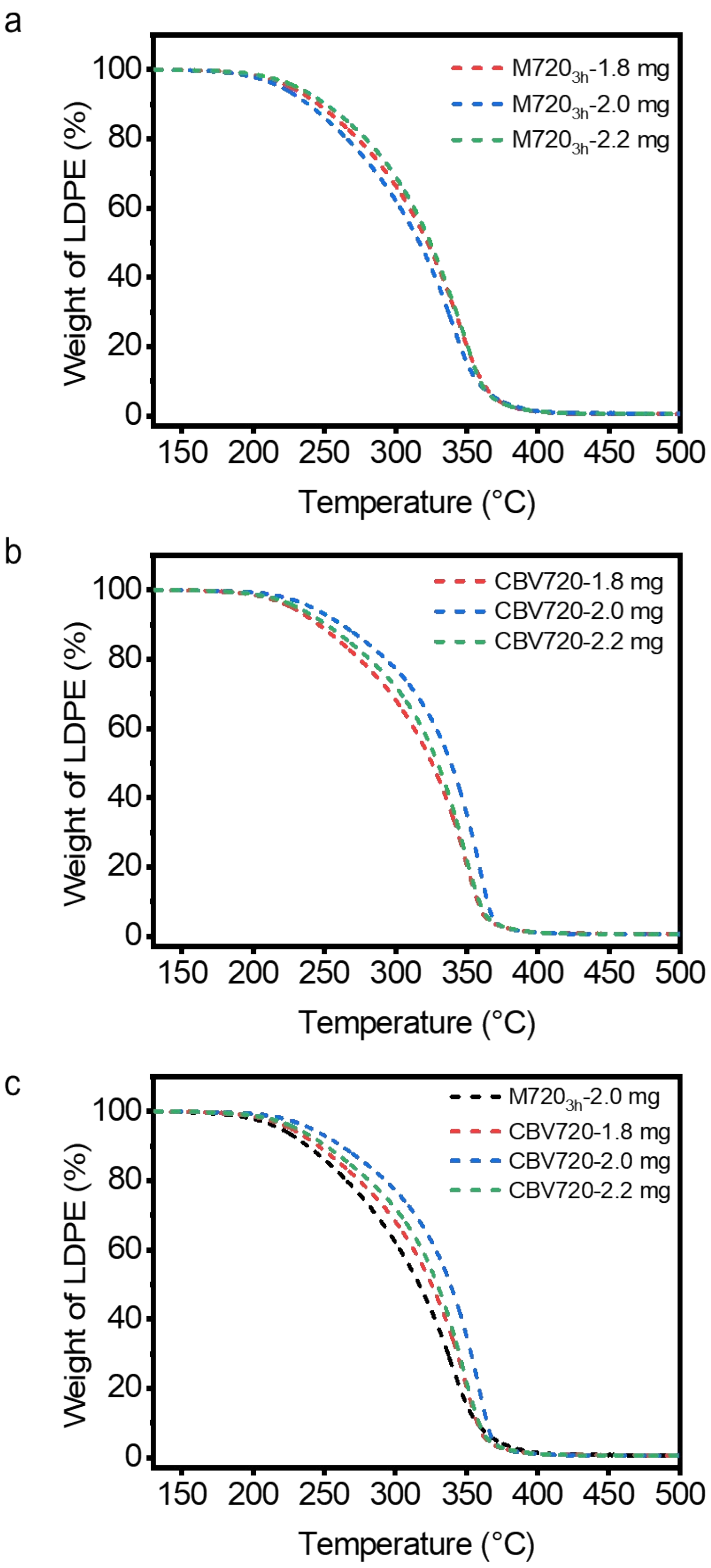

Figure S2. Weight loss curve of LDPE (6 mg) pyrolysis by varying the catalyst (CBV720 and M720$_{3h}$) amount to 1.8, 2.0 and 2.2 mg.

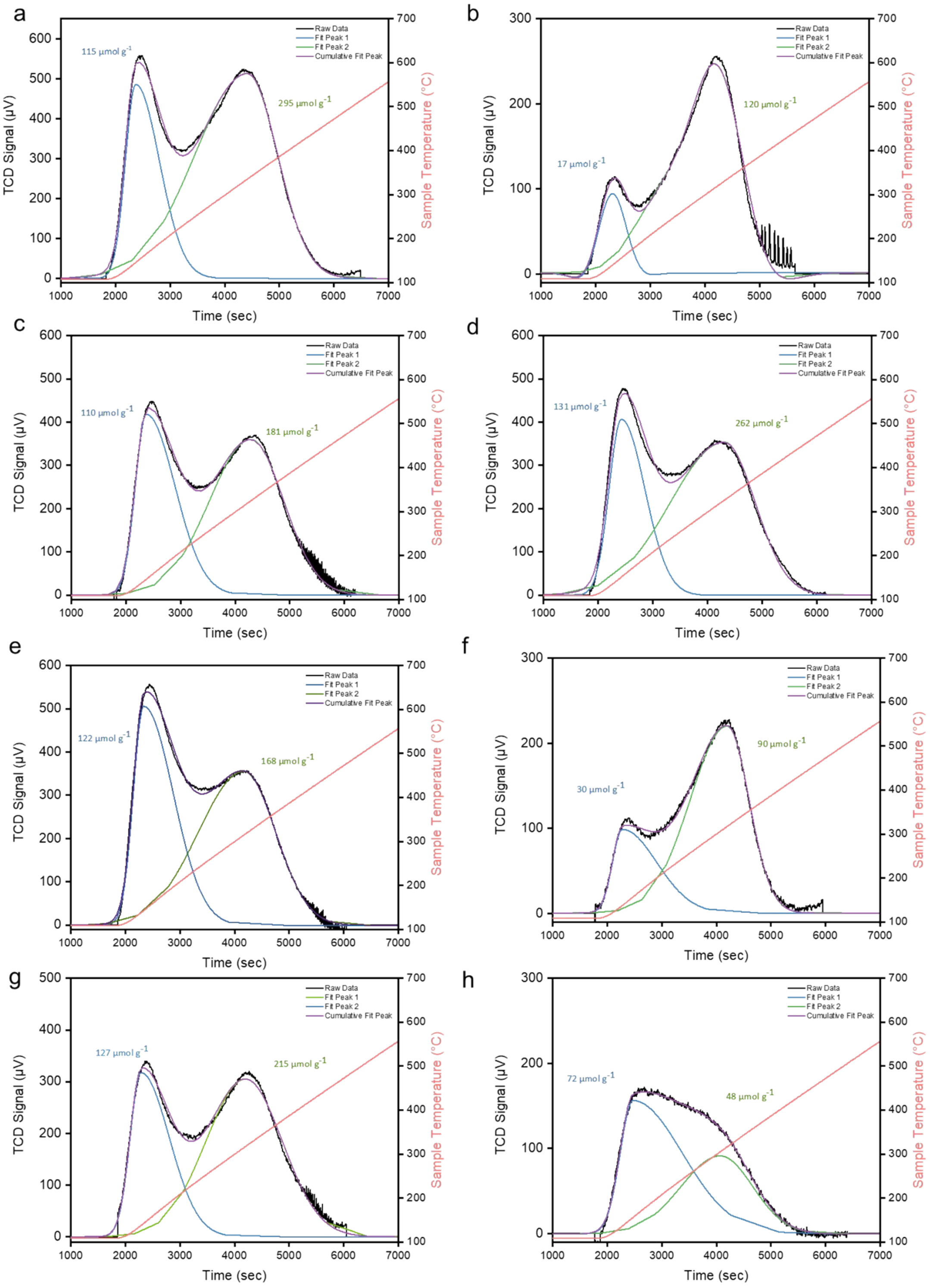

Figure S3 Ammonia TPD of (a) CBV720, (b) $CBV720_{HFSi}$, (c) $CBV720_{cit}$, (d) $M720_{3h}$, (e) $M720_{24h}$, (f) $M720_{HFSi}$, (g) $M720_{cit}$, (h) Al-MCM-41.

Table S1. T_{10} and T_{50} for degradation of HDPE, LLDPE and LDPE (6 mg) using different catalysts (2 mg).

S. No.	Catalyst	T_{10}(°C)	T_{50}(°C)
1.	Only HDPE	414	448
2.	CBV720	249.5	332.5
3.	$CBV720_{HFSi}$	256.5	337.5
4.	$CBV720_{cit}$	245.5	329
5.	$M720_{3h}$	241.5	322.5
6.	$M720_{HFSi}$	260.5	339
7.	$M720_{cit}$	235.5	312.5
8.	$M720_{24h}$	245.5	327.5
9.	Al-MCM-41	343.5	398.5

S. No.	Catalyst	T_{10}(°C)	T_{50}(°C)
1.	Only LLDPE	409	444
2.	CBV720	283.5	363.5
3.	$CBV720_{HFSi}$	313	375
4.	$CBV720_{cit}$	251	344.5
5.	$M720_{3h}$	288	362.5
6.	$M720_{HFSi}$	337	386.5
7.	$M720_{cit}$	281.5	360
8.	$M720_{24h}$	294.5	367
9.	Al-MCM-41	403	436

S. No.	Catalyst	T_{10}(°C)	T_{50}(°C)
1.	Only LDPE	392	442
2.	CBV720	263.5	349
3.	$CBV720_{HFSi}$	314.5	386
4.	$CBV720_{cit}$	253.5	352
5.	$M720_{3h}$	234	321
6.	$M720_{HFSi}$	256.5	340
7.	$M720_{cit}$	231	313.5
8.	$M720_{24h}$	240	325
9.	Al-MCM-41	323	381.5

Table S2. Different plastic wastes and their composition.

S. No.	Plastic wastes	Composition
1.	Dustbin	Polyethylene
2.	Lays Wrapper	Biaxially Oriented Polypropylene & LDPE
3.	Pepsi Bottle	Polyethylene terephthalate
4.	Milk Packet	Polyethylene
5.	Electric Waste	Polycarbonate
6.	PVC Pipes	Polyvinyl chloride
7.	Electric Wire	Polyvinyl chloride
8.	Food Packaging	Polyethylene, Polypropylene
9.	Bottle Cap	Polypropylene, high density polyethylene

[i]A. Chawla, A. Linares, J.D. Rimer, J. García-Martínez. Time-Resolved Dynamics of Intracrystalline Mesoporosity Generation in USY Zeolite. *Chem. Mater.* 2019, **31 (14),** 5005–5013.

[ii]A. Sachse, A. Grau-Atienza, E.O. Jardim, N. Linares, M. Thommes, J. García-Martínez. Development of Intracrystalline Mesoporosity in Zeolites through Surfactant-Templating. *Cryst. Growth Des.* 2017, **17 (8)**, 4289–4305.

[iii]M. J. Mendoza-Castro, E. Serrano, N. Linares, J. García-Martínez. Surfactant-Templated Zeolites: From Thermodynamics to Direct Observation. *Adv. Mater. Interfaces* 2021, **8 (4)**, 2001388.

[iv]I. Aracil, R. Font, J.A. Conesa. Semivolatile and volatile compounds from the pyrolysis and combustion of polyvinyl chloride. *J. Anal. Appl. Pyrol.* 2005, **74**, 465-478.

[v], J. Aguado, D. P. Serrano, J. M. Escola, A. Peral. Catalytic cracking of polyethylene over zeolite mordenite with enhanced textural properties. *J. Anal. Appl. Pyrolysis*, 2009, **85**, 352-358.
